# CGML++: Coarse-Grained Meta-Learning for Few-Shot Bearing Fault Detection Under Multiple Domain Shifts

**DOI:** 10.3390/s26216676

**Published:** 2026-10-22

**Authors:** Nikita Davydov, Ilya Repechko, Artem Nikonorov

**Affiliations:** AI Institute, Samara National Research University, Samara 443086, Russia; repechko@gmail.com (I.R.); artniko@gmail.com (A.N.)

**Keywords:** bearing faults detection, meta-learning, few-shot adaptation

## Abstract

Early detection of bearing defects in rotating machinery is crucial for preventing failures and reducing operational costs. However, deploying neural networks for vibration-based diagnostics is often hindered by a scarcity of labeled data under novel operating conditions. Existing adaptation techniques, including transfer learning and classical meta-learning algorithms, either suffer from negative transfer or fail to maintain sufficient accuracy under severe domain shifts. To address this, we propose CGML++ (Coarse-Grained Meta-Learning++), a novel few-shot method that aggregates MAML-trained domain experts through a tunable parameter *γ* combined with a transfer-learned model initialization, adapting to shift severity. We evaluated CGML++ on the CWRU bearing dataset under two domain shift scenarios: cross-sampling-rate transfer (12 kHz → 48 kHz) and cross-load transfer. In the 1-shot setting, CGML++ achieved a Macro F1-score of 0.88 and a Balanced Accuracy of 0.91 under cross-sampling-rate transfer, outperforming standard transfer learning by 0.18 and 0.12, respectively. Under cross-load transfer, CGML++ achieved a Balanced Accuracy of 0.96, surpassing all baselines by 0.04–0.09. The method’s versatility was validated across four architectures: ResNet1D, LightCNN, Transformer1D, and Informer1D. A key empirical finding is the identification of a phase transition at *γ*·*N* ≈ 2.0–3.5, enabling a scaling rule for adaptive hyperparameter tuning.

## 1. Introduction

Rotary machines—such as electric motors, turbines, compressors, and gearboxes—are fundamental to modern industrial infrastructure, energy, and transportation. Their reliable and uninterrupted operation is critical from both economic and safety perspectives. One of the most frequent and dangerous types of failure is the degradation of bearing components, which statistically accounts for 40–50% of all breakdowns in rotating equipment [1,2,3]. While more recent studies report figures in the range of 30–50% [4,5], there is a broad consensus that bearing faults remain the leading cause of failure in industrial rotating machinery [4,5,6,7]. The timely detection of incipient defects allows for the prevention of catastrophic accidents, the reduction of operational costs, and the effective implementation of condition-based maintenance (CBM) strategies [3,7].

Vibration analysis is widely recognized as the most informative and prevalent method for bearing condition monitoring [8,9]. In recent years, numerous review papers have comprehensively systematized the application of machine learning (ML) and deep learning (DL) techniques for processing vibration signals [6,10]. In particular, DL models have demonstrated superior performance in classifying bearing vibration patterns, achieving remarkable results even on benchmark datasets such as CWRU [11]. Beyond traditional fault diagnosis, vibration-based monitoring is also extensively utilized to evaluate bearing degradation in prognostics and health management (PHM) tasks, including remaining useful life (RUL) prediction [12,13].

In recent years, DL methods have revolutionized bearing fault diagnosis, offering a robust alternative to traditional methods that rely on manual feature extraction and domain expertise [2,10]. Unlike classical approaches, neural network architectures can automatically extract features from raw vibration signals, effectively adapting to nonlinear and non-stationary patterns [5,10]. Among various architectures, convolutional neural networks (CNNs) have become the predominant tool due to their ability to efficiently capture local spatial patterns from time series [14] and their time-frequency representations [5,11,15]. Numerous studies demonstrate that CNNs achieve exceptional classification accuracy on benchmark datasets, including CWRU [11,15]. For instance, modern lightweight multi-scale CNNs equipped with attention mechanisms have reported accuracies exceeding 99% on the CWRU dataset for both single and combined bearing faults [15]. Similarly, meta-optimized CNNs have achieved average accuracies of 99.91% [16], while hybrid CNN-BiLSTM architectures have shown high efficacy under limited sample conditions [17].

Parallel to the evolution of CNNs, Transformer architectures have gained significant traction. Leveraging the self-attention mechanism [18], Transformers and their variants, including Informer, have been increasingly adopted for vibration-based fault diagnosis due to their ability to capture long-range temporal dependencies [19,20,21]. In bearing diagnostics, they have proven highly effective, particularly for tasks involving long time series and complex time-frequency patterns [20,21]. Furthermore, hybrid CNN-Transformer architectures, which synergize the local feature extraction of CNNs with the global dependency modeling of Transformers, have demonstrated superior performance. For example, such hybrids have achieved an average accuracy of 96.25% across five benchmark datasets, outperforming standard Transformers by 8.6% [20]. Other hybrid models, such as the EMD-CNN-Transformer, have also consistently surpassed standalone Transformers, CNN-LSTMs, and standard CNN-Transformers on the CWRU dataset [21].

More recently, the emergence of Large Language Models (LLMs) has opened new avenues for intelligent fault diagnosis. For instance, Xu et al. [22] proposed an adaptive industrial LLM for mechanical fault diagnosis under variable operating conditions, leveraging the natural language understanding capabilities of LLMs to interpret diagnostic knowledge and adapt to changing operating regimes. While LLM-based approaches show promise for knowledge-intensive diagnostic tasks, they currently require substantial computational resources and are primarily applicable to tasks involving textual or structured diagnostic data. For vibration-based anomaly detection with raw time-series inputs and extreme data scarcity (1-shot), parameter-efficient meta-learning methods such as the one proposed in this work remain more practical.

Despite the remarkable success of deep learning, its practical deployment in bearing diagnostics faces a fundamental bottleneck: high accuracy is heavily contingent upon the availability of massive amounts of labeled data [10,23]. In real industrial environments, collecting and annotating vibration data for every new machine type, operating regime, or fault condition is prohibitively expensive and labor-intensive [1,2]. Furthermore, mechanical systems cannot be intentionally operated with severe defects for extended periods, making the acquisition of diverse fault data particularly challenging [11]. This discrepancy between laboratory benchmarks (often exceeding 99% accuracy) and practical performance under data-scarce conditions strongly motivates the development of few-shot learning (FSL) [24] and meta-adaptation techniques [5,10,25]. Recent advances in few-shot learning for industrial fault diagnosis include attention-enhanced meta-learning networks [26] and semi-supervised approaches that leverage unlabeled data. Compounding this data scarcity is the challenge of domain shift—the alteration of statistical signal properties when transitioning between different operating conditions [2,5,11]. Models trained on a specific source domain often suffer a severe performance drop when applied to target data collected under different conditions [11].

The Case Western Reserve University (CWRU) bearing dataset is the most widely used benchmark for studying domain shift in this field [8,10]. A key feature of the CWRU dataset, which makes it highly valuable for evaluating robust cross-domain adaptation, is the presence of two distinct sources of variability: varying mechanical loads (and consequently, rotational speeds) and different sensor sampling rates (12 kHz and 48 kHz) [4,8]. While adapting across different loads introduces a standard domain shift by altering vibration amplitudes and frequency characteristics [3,8], transitioning between sampling rates (e.g., 12 kHz → 48 kHz) represents a structurally severe sensor-level domain shift. This quadruples the temporal resolution, capturing higher-frequency vibration components, but fundamentally alters the signal representation and statistical distribution, creating one of the most challenging scenarios in vibration analysis [5,10].

To address domain shift under few-shot conditions, several approaches have been proposed. Transfer learning methods adapt a pre-trained source model to the target domain via fine-tuning [2,5]; however, under severe domain shifts, they often suffer from catastrophic forgetting or negative transfer [5]. Domain adversarial training methods, such as DANN-based approaches [27], have been successfully applied to cross-domain fault diagnosis by learning domain-invariant features. Meta-learning algorithms, such as Model-Agnostic Meta-Learning (MAML) [28] and its variants [29,30], seek an optimal parameter initialization that allows rapid adaptation to a new domain within one or a few gradient steps [28,30]. Recently, hybrid frameworks combining domain adaptation and meta-learning have gained traction to enhance robustness. For instance, the DBDA-FD framework aligns feature distributions at global and sub-domain levels, achieving 97.6% accuracy on CWRU in a five-way five-shot setting [31]. Similarly, the AdaMETA framework combines dynamic MAML with multi-scale attention, reaching up to 99.26% accuracy in cross-load scenarios [32]. However, these methods operate under moderate cross-load shifts at a fixed sampling rate. As demonstrated in our experiments, most existing methods fail to maintain sufficient accuracy when confronted with the simultaneous challenge of a structurally severe sensor-level domain shift (12 kHz → 48 kHz) and extreme data scarcity (1-shot learning).

To address these limitations, in this paper, we propose CGML++ (Coarse-Grained Meta-Learning++), a novel domain expert aggregation method for few-shot binary anomaly classification under severe domain shifts. Building upon the foundational concept of coarse-grained meta-learning [33], our approach introduces three key enhancements. First, instead of training a single meta-model, we train multiple MAML-based experts on diverse subsets of the source domain (e.g., different load conditions in the CWRU dataset). Each expert specializes in its respective sub-domain, which preserves knowledge diversity and mitigates the gradient conflicts typical of standard MAML when handling heterogeneous data [5,28]. Second, we introduce a parameterized aggregation mechanism: the parameters of the MAML experts are first averaged, and this aggregated vector is then linearly combined with the initialization from a large-scale transfer model using a tunable parameter *γ*. Consequently, the final initialization represents a weighted combination of the averaged experts and the transfer model, where *γ* explicitly regulates the influence of the meta-learned experts. Third, this large-scale transfer model is trained on the same source domains as the experts using standard transfer learning. This ensures a stable training start and prevents the divergence that sometimes occurs with purely meta-learned initializations [28,30], effectively synergizing the robustness of transfer learning with the adaptability of meta-learning.

Furthermore, a crucial feature of CGML++ is its architectural universality. The method has been implemented on top of three distinct architectures—ResNet1D, LightCNN, and Transformer1D—demonstrating its model-agnostic nature and making it applicable to a wide range of time-series diagnostic tasks. We conducted an extensive experimental study on the CWRU dataset, focusing on the most challenging scenario: the cross-sampling-rate shift (12 kHz → 48 kHz). A key empirical finding of this work is the identification of a phase transition when varying the parameter *γ*: classification performance improves sharply once the threshold *γ*·*N* ≈ 2.0–3.5 is reached (where *N* is the number of experts). This phenomenon allows us to formulate a novel scaling rule, indicating that the optimal value of *γ* depends on the product *γ*·*N*, rather than on each parameter independently.

The main contributions of this paper are summarized as follows:

- Novel Meta-Learning Architecture: We propose CGML++, a few-shot learning framework that aggregates multiple MAML-trained domain experts via a tunable parameter *γ* and a transfer-learned initialization, specifically designed to handle severe sensor-level domain shifts.
- Robust Performance across Multiple Domain Shifts: We demonstrate that CGML++ significantly outperforms standard transfer learning, MAML, Reptile, and FOMAML under both a severe sensor-level shift (12 kHz → 48 kHz cross-sampling-rate) and a cross-load shift (HP 0–1–2 → HP 3), confirming robustness across fundamentally different types of domain shift.
- Empirical Discovery of a Phase Transition: We identify a critical phase transition at *γ*·*N* ≈ 2.0–3.5 and derive an empirical scaling rule for adaptive hyperparameter tuning, eliminating the need for exhaustive grid searches in practical industrial deployments.
- Architectural Agnosticism: We validate the versatility of the proposed method across four fundamentally different backbones (ResNet1D, LightCNN, Transformer1D and Informer1D), confirming its broad applicability to various vibration-based condition monitoring tasks.

The remainder of this paper is organized as follows: Section 2 details the proposed CGML++ methodology and the experimental setup. Section 3 and Section 4 present and discuss the experimental results. Finally, Section 5 concludes the paper.

## 2. Materials and Methods

### 2.1. Coarse-Grained Meta-Learning: General Concept

Meta-learning algorithms, particularly Model-Agnostic Meta-Learning (MAML) [28], aim to find a model initialization $\theta_{0}$ that enables rapid adaptation to novel tasks $T_{k}$ with only a few gradient steps. In the standard MAML formulation, the meta-update aggregates task-specific gradients across a batch of tasks:

(1) $$\theta_{0}\leftarrow \theta_{0}-\beta \sum_{k=1}^{K}\nabla_{\theta }L_{T_{k}}\left(\theta_{k}^{\prime }\right),$$

where $\theta_{k}^{\prime }$ denotes the adapted parameters after inner-loop updates on task $T_{k}$, and *β* is the meta-learning rate. However, when tasks originate from heterogeneous domains (e.g., different sensor configurations or operating loads), this fine-grained gradient aggregation suffers from two fundamental issues: gradient conflicts between tasks with divergent loss landscapes, and training instability under severe domain shifts [5,28]. Alternative approaches to handling domain shift include correlation alignment methods such as CORAL [34], which minimize the discrepancy between source and target feature covariance matrices.

To address these limitations, the concept of coarse-grained meta-learning was introduced in our prior work [33]. Instead of averaging gradients during the meta-update, coarse-grained meta-learning operates at the parameter level: each expert is independently trained on its respective sub-domain, and the final initialization is obtained by averaging the resulting weight vectors. Formally, given *N* sub-domains $\left\{D_{1},\;\ldots ,\;D_{N}\right\}$, we train *N* independent MAML experts $\left\{\theta_{1}^{*},\;\ldots ,\;\theta_{N}^{*}\right\}$ and compute the aggregated initialization as:

(2) $$\bar{\theta }=\frac{1}{N}\sum_{i=1}^{N}\theta_{i}^{*}.$$

This strategy offers two key advantages. First, by decoupling the training of individual experts, it eliminates inter-task gradient interference entirely. Second, it preserves the diversity of domain-specific knowledge encoded in each expert’s parameters. The resulting averaged model *θ* lies in a flat region of the loss landscape—a property related to linear mode connectivity [35]—and serves as a robust, generalizable initialization for few-shot adaptation on unseen target domains.

The coarse-grained meta-learning procedure can be summarized as follows:

1. Partition the source domain into *N* sub-domains $\left\{D_{1},\;\ldots ,\;D_{N}\right\}$ (e.g., by operating load or sensor configuration).
2. Train *N* independent MAML experts, each specialized on its sub-domain $D_{I}$, yielding parameters $\left\{\theta_{1}^{*},\;\ldots ,\;\theta_{N}^{*}\right\}$.
3. Aggregate the expert parameters via uniform averaging to obtain *θ*.
4. Adapt *θ* to the target domain $D_{\mathrm{target}}$ using a few gradient steps on the support set.

While effective under moderate domain shifts, this vanilla formulation treats all experts equally and does not account for the degree of shift between the source and target domains.

### 2.2. Mathematical Derivation from MAML

We now derive the coarse-grained aggregation formula starting from the standard MAML formulation, following the approach introduced in [33]. Consider *N* source sub-domains $\left\{D_{1},\;\ldots ,\;D_{N}\right\}$, each with its own data distribution. The MAML algorithm operates in two nested loops.

For each sub-domain i, the model parameters are updated via *K* gradient steps on the support set:

(3) $$\theta_{i}^{\left(k+1\right)}=\theta_{i}^{\left(k\right)}-\alpha \;\nabla_{\theta }L_{i}\;\left(\theta_{i}^{\left(k\right)}\right),k=0,\ldots ,K-1,$$

where *α* is the inner learning rate and $L_{i}$ is the task-specific loss. After *K* steps, the adapted parameters for sub-domain *i* are denoted $\theta_{i}^{*}\equiv \theta_{i}^{\left(K\right)}$.

The meta-learner updates the shared initialization *θ* by aggregating task-level gradients:

(4) $$\theta \leftarrow \theta -\beta \sum_{i=1}^{N}\nabla_{\theta }L_{i}\;\left(\theta_{i}^{*}\right),$$

where *β* is the meta-learning rate. Computing this gradient exactly requires second-order derivatives (the Hessian), which is computationally prohibitive for deep networks. Following the first-order approximation (FOMAML) [28], and noting that for ReLU-activated networks the second derivatives are near-zero almost everywhere the meta-update simplifies to:

(5) $$\theta^{\left(t+1\right)}=\theta^{\left(t\right)}-\beta \sum_{i=1}^{N}\nabla_{\theta }L_{i}\left(\theta_{i}^{*}\right)$$

The key algebraic insight of coarse-grained meta-learning is that the cumulative gradient sum in (5) can be expressed in terms of the parameter displacements of individual experts. After *K* inner-loop steps, the total displacement of expert *i* from the shared initialization is:

(6) $$\theta_{i}^{*}-\theta =-\alpha \sum_{k=0}^{K-1}\nabla_{\theta }L_{i}\left(\theta_{i}^{\left(k\right)}\right)$$

Averaging (6) over all Nexperts and rearranging the identity:

(7) $$\frac{\alpha }{N}\sum_{i=1}^{N}\sum_{k=0}^{K-1}\nabla_{\theta }L_{i}\left(\theta_{i}^{\left(k\right)}\right)=\theta -\frac{1}{N}\sum_{i=1}^{N}\theta_{i}^{*}$$

It is important to note that the substitution of Equation (7) into Equation (5) constitutes a motivated approximation rather than a strict algebraic identity. In the exact MAML formulation, the meta-gradient is computed with respect to the query-set loss, whereas Equation (7) captures the parameter displacement induced by the support-set gradients. However, under the first-order approximation and assuming that the support and query sets are drawn from the same sub-domain distribution, the direction of the inner-loop displacement aligns with the query-loss gradient. This alignment assumption is standard in FOMAML-type derivations [25] and is empirically validated by the consistent performance gains observed in Section 3. We emphasize that the final aggregation formula (Equation (10)) is subsequently promoted to a free hyperparameter *γ*, which absorbs any residual approximation error and allows the method to be calibrated empirically.

Substituting (7) into the cumulative form of (5) over multiple meta-iterations, the updated parameters can be written as a convex combination of the original initialization and the averaged expert parameters:

(8) $$\theta_{\mathrm{new}}=\left(1-\frac{\beta }{\alpha }N\right)\theta +\frac{\beta }{\alpha }\sum_{i=1}^{N}\theta_{i}^{*}$$

The special case $\frac{\beta }{\alpha }=\frac{1}{N}$ reduces (4) to pure uniform averaging:

(9) $$\bar{\theta }=\frac{1}{N}\sum_{i=1}^{N}\theta_{i}^{*},$$

which defines the vanilla coarse-grained meta-learning (CGML) formulation [33].

In the original theoretical derivation (vanilla CGML [29]), the interpolation coefficient was implicitly tied to the ratio of learning rates (*β*/*α*). In CGML++, we decouple this rigid relationship and promote the coefficient to a free hyperparameter *γ*, making the effective step size *γ*·*N* independent of the training hyperparameters. This decoupling provides greater flexibility: *γ*·*N* is the practical, useful formulation for hyperparameter selection, while *β*/*α* was merely the theoretical starting point that motivated the aggregation approach.

The interpolation coefficient *γ*·*N* is introduced to dynamically balance two competing sources of knowledge: the global, generalized features from the transfer-learned initialization *θ*_init_, and the local, domain-specific features from the averaged experts *θ*_experts_. Without this coefficient (i.e., with *γ*·*N* = 1, pure averaging), the method may either under-utilize the robust transfer initialization (if experts are poorly trained) or under-weight the domain-specific expert knowledge (if the domain shift is severe). The tunable coefficient allows the method to adapt the interpolation strength based on the severity of the domain shift, as empirically validated in Section 3.3.

In the original derivation, the interpolation coefficient $\frac{\beta }{\alpha }$ is implicitly fixed by the choice of learning rates. We observe that this coefficient need not be constrained to the single value $\frac{1}{N}$. Instead, we promote it to a free hyperparameter *γ*, yielding the generalized aggregation formula:

(10)  θfinal=1−γNθinit+γN·θ¯experts 

where:

- $\theta_{\mathrm{init}}$ is a pre-trained initialization;
- ${\bar{\theta }}_{\mathrm{experts}}=\frac{1}{N}\sum_{i=1}^{N}\theta_{i}^{*}$ is the averaged expert vector from (5);
- *γN* is the interpolation coefficient.

where *γ* ≥ 0 is a free hyperparameter and *γN* is the effective step size along the direction from $\theta_{\mathrm{init}}$ toward ${\bar{\theta }}_{\mathrm{experts}}$. We deliberately do not constrain *γN* ≤ 1: the formula is well-defined for *γN* > 1, in which case it extrapolates beyond the averaged experts.

This yields three regimes:

- *γN* < 1—interpolation between $\theta_{\mathrm{init}}$ and ${\bar{\theta }}_{\mathrm{experts}}$;
- *γN* = 1—$\theta_{\mathrm{final}}={\bar{\theta }}_{\mathrm{experts}}$, recovering vanilla CGML [32];
- *γN* > 1—extrapolation past the experts. As shown in Section 3, the optimum lies in regime (3), which is precisely why CGML++ outperforms vanilla CGML: uniform expert averaging alone under-shoots the optimal initialization.

### 2.3. CGML++: Modifications and Full Procedure

The vanilla CGML formulation [33] employs a fixed aggregation strategy: all N experts are uniformly averaged, and the result is directly used as the initialization for few-shot adaptation. While this improves upon standard MAML by eliminating gradient conflicts, it lacks flexibility in two important aspects: (1) the relative influence of the expert ensemble is fixed at γN=1 (pure averaging), with no mechanism to adjust it based on the characteristics of the target domain; and (2) the initialization is entirely determined by the experts, with no provision for incorporating an independently trained global model. Compared to the vanilla CGML formulation [29], CGML++ introduces three key modifications: (1) parametrized aggregation via a free hyperparameter *γ* instead of a fixed coefficient; (2) flexible initialization *θ*_init_ from a transfer-learned model; (3) generalization to an arbitrary number of experts N. These modifications are detailed below.:

Modification 1: Parametrized aggregation via γ. Instead of the fixed coefficient $\frac{\beta }{\alpha }=\frac{1}{N}$ inherited from the MAML learning rates, we introduce a free hyperparameter γ≥ 0 that explicitly controls the interpolation strength between the transfer initialization and the expert ensemble (Equation (10)). This decouples the aggregation weight from the training hyperparameters and allows systematic exploration of the interpolation spectrum—from pure transfer learning (γ=0) to pure expert averaging ($\gamma =\frac{1}{N}$). As shown in Section 3, the optimal operating point is governed by the product γ·N rather than by γ alone, revealing a scaling invariance that simplifies hyperparameter selection in practice.

Modification 2: Flexible initialization $\theta_{\mathrm{init}}$. In the vanilla version, the initialization is implicitly defined by the meta-learning process. In CGML++, $\theta_{\mathrm{init}}$ can be any pre-trained model. In this work, $\theta_{\mathrm{init}}$ is a transfer model trained via standard supervised learning on all source domains combined. This provides a stable, well-generalized starting point that prevents the training divergence occasionally observed with purely meta-learned initializations [28,30], while the expert term ${\bar{\theta }}_{\mathrm{experts}}$ injects domain-specific adaptability.

Modification 3: Generalization to arbitrary N. The aggregation formula (Equation (10)) is defined for any number of experts N≥1. In this work, we set N=12, corresponding to all combinations of 4 operating loads (0, 1, 2, 3 HP) and 3 defect severities (diameters 0.007, 0.014, 0.021 inches) available in the CWRU dataset at 12 kHz. However, the method imposes no restriction on this configuration and can accommodate any partitioning of the source domain.

**Figure 1:**
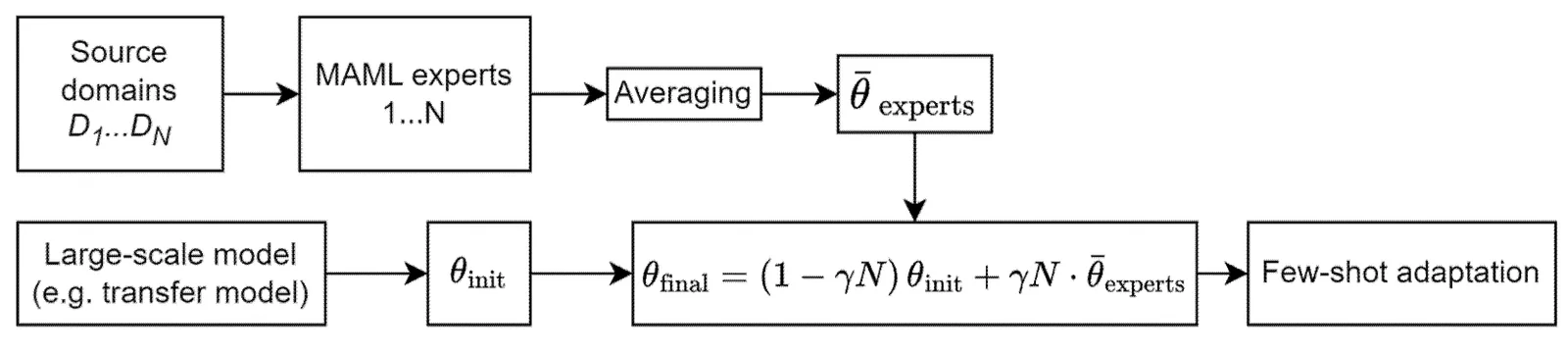
Block diagram of the proposed CGML++ method. N=12 domain-specific MAML experts are independently trained on their respective source sub-domains (combinations of load and defect severity at 12 kHz). Their weight vectors are uniformly averaged to produce ${\bar{\theta }}_{\mathrm{experts}}$, which is then linearly combined with a global transfer initialization $\theta_{\mathrm{init}}$ via the tunable parameter γ, yielding the final initialization $\theta_{\mathrm{final}}$. This initialization is subsequently fine-tuned on the target domain (48 kHz) using a 1-shot support set.

The whole algorithm is shown in the table Algorithm 1 and on the Figure 1.
**Algorithm 1:** CGML++—Training, Aggregation, and Adaptation**Input:**
Source sub-domains:
$D_{1},D_{2},\ldots ,D_{N}$(N=12)
Target domain:
$D_{\mathrm{target}}$ (48 kHz)
Inner learning rate:
α
Meta-learning rate:
β
Adaptation learning rate:
$\alpha^{\prime }$
Number of inner-loop steps:
K
(K=5)
Number of meta-iterations:
M
(M=10)
Tunable interpolation parameter:
γ≥0
              
(tunable; optimum at γN ≈ 2.0–3.5)
Pre-trained initialization:
$\theta_{\mathrm{init}}$ (transfer model)**Phase 1: Training Domain-Specific MAML Experts**For each sub-domain *i* = 1 to *N*:Initialize expert parameters: *θ_i_* ← *θ*_init_For meta-iteration t = 1 to *M*:Sample a batch of *B* tasks {𝒯*_j_*} from 𝒟*_i_*For each task 𝒯*_j_**Set θ_i′_ ←θ_i_**Inner loop (support set adaptation):**for k = 1 to K:**θ_i′_* ← *θ_i′_* − *α* $\nabla_{\theta }$, ℒ*_sup_(𝒯_j_, θ_i′_)*Compute query loss: *ℓ_j_ ←* ℒ*_qry_(𝒯_j_, θ_i′_)*Meta-update:*θ_i′_* ← *θ_i_* − *(β/B)* $\sum_{j=1}^{B}\nabla_{\theta }l_{j}$Store trained expert: θ_i*_**Phase 2: Coarse-Grained Aggregation** Compute averaged expert vector:${\bar{\theta }}_{\mathrm{experts}}$*=*$\frac{1}{N}\sum_{i=1}^{N}\theta_{i}^{*}$Compute final initialization (Equation (10)):$\theta_{\mathrm{final}}$ = $\left(1-\gamma N\right)\theta_{\mathrm{init}}+\gamma N\cdot {\bar{\theta }}_{\mathrm{experts}}$**Phase 3: Few-Shot Adaptation on Target Domain**Sample 1-shot support set from 𝒟_target:𝒮 = {(x_norm_, 0), (x_anom_, 1)}Fine-tune on support set:*θ*_adapted_ = *θ*_final_ − *α*′ *∇θ*_final_ ℒ(𝒮, *θ*_final_)Evaluate *θ*_adapted_ on query set 𝒬 ⊂ 𝒟_target_

### 2.4. Description of Dataset: CWRU Bearing Dataset

**Figure 2:**
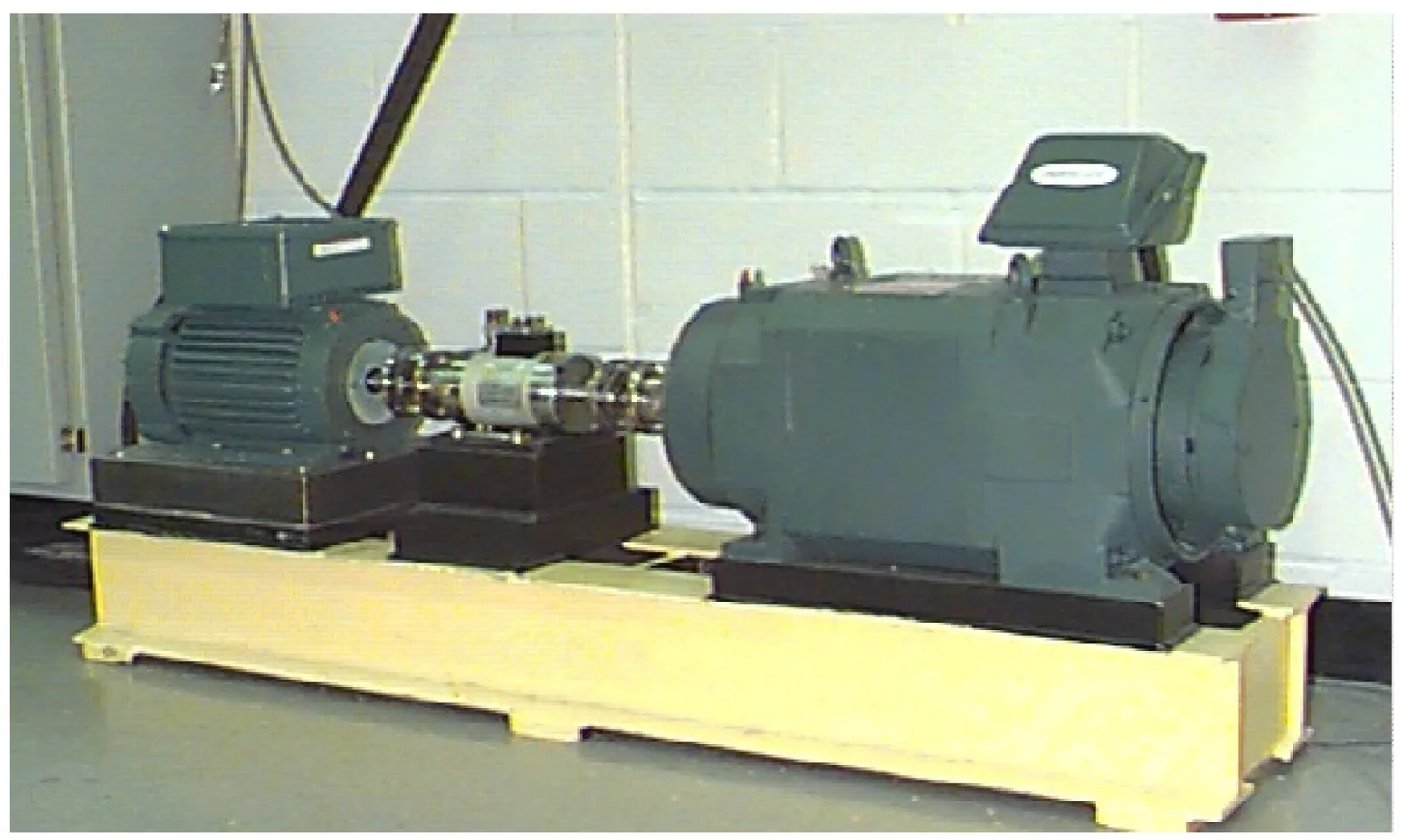
Experimental test rig of the CWRU bearing dataset. The drive-end bearing (SKF 6205) and fan-end bearing (SKF 6203) are instrumented with accelerometers mounted on the motor housing.

We evaluate the proposed method on the publicly available Case Western Reserve University (CWRU) bearing dataset [7,8,36], which is widely used as a benchmark for bearing fault diagnosis research. The dataset was collected from a motor–torque transducer–dynamometer test rig equipped with a 2 hp (1.49 kW) three-phase induction motor. Two deep-groove ball bearings were instrumented: an SKF 6205 at the drive end and an SKF 6203 at the fan end [7]. The mechanical load is applied radially to the shaft via the dynamometer, which translates to a radial load on both bearings. This loading configuration is typical for deep-groove ball bearings, which are designed primarily to support radial loads with limited axial load capacity. Vibration signals were acquired using accelerometers mounted on the motor housing. Example of experimental test rig of CWRU bearing dataset is shown on Figure 2.

The dataset encompasses four bearing health states: (i) normal (healthy) condition, (ii) inner race fault, (iii) outer race fault, and (iv) ball (rolling element) fault. For each fault type, defects of varying severity were introduced via electro-discharge machining with diameters of 0.007, 0.014, 0.021, and 0.028 inches (0.178, 0.356, 0.533, and 0.711 mm) [7,8]. For the SKF 6205 bearing used in the drive end (bore diameter 25 mm, outer diameter 52 mm, ball diameter 7.94 mm), these defect sizes represent incipient to moderate localized faults on the raceways, ranging from approximately 2% to 9% of the ball diameter. Such defect sizes are representative of early-stage faults that are challenging to detect but critical for predictive maintenance. In this work, we adopt a binary anomaly detection formulation: all three fault types across all available severities are grouped into a single “anomalous” class, while the healthy condition constitutes the “normal” class. This binary formulation is chosen because (i) it reflects the primary industrial need—detecting the presence of any fault before scheduling detailed diagnostics—and (ii) it enables a controlled evaluation of the meta-learning framework without confounding effects from inter-class confusion.

Experiments were conducted under four motor loads—0, 1, 2, and 3 hp—corresponding to rotational speeds of 1797, 1772, 1750, and 1730 RPM, respectively [4,7]. While “hp” (horsepower) denotes the motor’s power rating rather than a direct mechanical load unit (e.g., torque in N·m), it is the standard nomenclature used in the CWRU dataset documentation to denote the operating condition. This load variation introduces a natural domain shift, as vibration amplitudes and spectral characteristics change with the operating condition [7].

Vibration signals were recorded at two sampling rates: 12 kHz and 48 kHz. In this study, we use the drive-end accelerometer data, which are available at both sampling rates for all four loads. The 48 kHz data provide four times higher temporal resolution than the 12 kHz data, capturing broader high-frequency vibration components but fundamentally altering the signal representation. In our experiments, the 12 kHz recordings serve as the source domain and the 48 kHz recordings as the target domain, constituting a challenging cross-sampling-rate transfer scenario.

We exclude the 0.028-inch defect severity from our analysis for two reasons: (i) several fault types are missing at this diameter in the original dataset, resulting in incomplete class coverage; and (ii) the corresponding 48 kHz recordings are not available, making cross-sampling-rate evaluation impossible. The remaining three defect diameters (0.007, 0.014, and 0.021 inches) are available at both sampling rates for all four loads and all three fault types.

Based on the available data, we define N=12 source sub-domains for expert training, corresponding to all combinations of 4 operating loads (0, 1, 2, 3 hp) and 3 defect diameters (0.007, 0.014, 0.021 inches) at 12 kHz. Each sub-domain contains vibration samples from all three fault types (inner race, outer race, ball) at the corresponding severity level, along with normal samples recorded under the same load condition. The target domain comprises the corresponding 48 kHz recordings under identical load and defect configurations.

For signal preprocessing, the raw vibration time series are segmented into non-overlapping windows of 1024 samples, corresponding to approximately 85 ms at 12 kHz and 21 ms at 48 kHz. Each window is treated as an independent sample and labeled according to the original data annotation (normal or anomalous). Z-score normalization is applied to each segment individually (zero mean, unit variance) to ensure consistent scaling and improve training stability. No additional preprocessing (e.g., band-pass filtering, detrending, or envelope analysis) is applied, in order to preserve the raw spectral characteristics of the vibration signals.

### 2.5. Domain Shift Quantification

To quantitatively assess the severity of the domain shifts considered in this study, we compute three complementary metrics between the source and target distributions:

1. The proxy A-distance [37], defined as d_A_ = 2(1 − 2ε), where ε is the test error of a linear SVM trained to distinguish source from target samples. The A-distance ranges from 0 (indistinguishable domains) to 2 (perfectly separable domains).
2. The Maximum Mean Discrepancy (MMD) [37] with a Gaussian kernel and median heuristic for bandwidth selection, measuring the distance between source and target feature distributions in the representation space of the pre-trained transfer model.
3. The spectral distance, computed as the mean Euclidean distance between L2-normalized power spectral densities of source and target vibration signals, restricted to the common frequency band.

The results are summarized in Table 1. The cross-frequency shift (12 kHz → 48 kHz) exhibits the highest values across all three metrics (A-distance = 1.949, MMD = 0.189, spectral distance = 1.322), confirming it as the most severe domain shift available in the CWRU benchmark. Among the four cross-load configurations, the shift severity varies considerably (MMD ranges from 0.026 to 0.151), with HP0 and HP3 as target domains producing the largest shifts, consistent with their position at the extremes of the load range. Notably, even the most severe cross-load configuration (HP 0+1+2 → HP 3, MMD = 0.151) remains below the cross-frequency shift (MMD = 0.189), which involves a fundamental change in signal representation rather than a mere rescaling of vibration amplitudes.

The MMD values reported in Table 1 are consistent with findings in the domain adaptation literature [38,39], where MMD > 0.15 typically indicates a challenging transfer scenario requiring specialized adaptation methods.

## 3. Results

### 3.1. Experimental Setup

We evaluate CGML++ on the CWRU dataset under the challenging cross-sampling-rate scenario (12 kHz → 48 kHz). The source domain consists of N=12 sub-domains (4 loads × 3 defect diameters), each used to train a separate MAML expert. The target domain comprises the corresponding 12 sub-domains recorded at 48 kHz. The task is binary anomaly detection: normal vs. anomalous (all fault types combined). In the 1-shot setting, each support set contains one normal and one anomalous sample, while the query set contains 116 samples (58 per class). All results are averaged over 15 independent runs on each of the 12 target sub-domains, with standard deviations reported.

To demonstrate the architectural agnosticism of CGML++, we evaluate it on three fundamentally different backbones spanning two orders of magnitude in model capacity:

- ResNet1D—a 1D residual network comprising an initial convolutional layer (kernel 7, stride 2, 64 channels), a max-pooling layer, and four residual stages with 2 blocks each (64 → 128 → 256 → 512 channels), followed by global average pooling and a linear classifier. The architecture contains approximately 2.8 million parameters and relies on skip connections to facilitate gradient flow in deep time-series models.
- LightCNN—a lightweight 1D convolutional network consisting of three sequential convolutional blocks (16 → 32 → 64 channels, kernels 7/5/3, each followed by batch normalization, ReLU activation, and pooling), terminated by adaptive average pooling and a linear classifier. With only ~50 thousand parameters, LightCNN is designed for resource-constrained industrial edge devices.
- Transformer1D—a 1D Transformer encoder with sinusoidal positional encoding, a 1D convolutional input projection (1 → 128 channels), and 4 encoder layers (d_model = 128, 8 attention heads, feedforward dimension 512, GELU activation, dropout 0.1). Global mean pooling over the temporal axis precedes the linear classifier. The model contains approximately 2.5 million parameters and captures long-range temporal dependencies via self-attention.
- Informer—a ProbSparse self-attention Transformer specifically designed for efficient time-series processing [40]. The architecture comprises a 1D convolutional input projection (stride 4), sinusoidal positional encoding, 2 encoder layers with ProbSparse attention and convolutional distilling (halving the sequence length at each layer), followed by global average pooling and a linear classifier. With approximately 0.94 million parameters, Informer occupies an intermediate capacity range between LightCNN (50K) and Transformer1D (2.5M). Unlike the standard Transformer1D, Informer employs ProbSparse attention with O(L·log L) complexity and convolutional distilling, representing a fundamentally different attention mechanism.

All four architectures accept raw 1024-sample vibration windows as input and output a binary prediction (normal/anomalous). The three orders of magnitude difference in parameter count between LightCNN (50K) and ResNet1D (2.8M), combined with two distinct attention mechanisms (full attention in Transformer1D and ProbSparse attention in Informer), provides a stringent test of the method’s universality.

To evaluate the generalizability of CGML++ across different types of domain shift, we additionally conduct experiments under a cross-load transfer scenario. In this setting, the source domain comprises sub-domains from three operating loads at 12 kHz, and the target domain corresponds to the remaining load at the same sampling rate. All other experimental conditions (architecture, adaptation procedure, evaluation metrics) are identical to the cross-sampling-rate experiments.

We compare CGML++ against the following methods:

- Transfer Learning (Transfer): A model pre-trained on all source domains via standard supervised learning, fine-tuned on the target support set. No meta-learning is involved.
- MAML [28]: Model-Agnostic Meta-Learning with second-order gradients. The model is meta-trained across all source sub-domains and adapted to the target domain.
- FOMAML [28]: First-order approximation of MAML, which drops the Hessian computation for efficiency.
- Reptile [30]: A gradient-free meta-learning algorithm that updates the initialization by moving toward the task-specific parameters.

All baselines use the same architectures, support/query splits, and number of adaptation steps as CGML++ to ensure a fair comparison.

Hyperparameters. Table 2 summarizes the key hyperparameters used across all experiments. No hyperparameter tuning was performed on the target-domain query set.

The inner learning rate *α* controls the step size during task-specific adaptation in the inner loop. It must be sufficiently large to allow meaningful parameter updates within *K* = 5 steps, typically falling in the range [0.01, 0.1]. Values below 0.01 lead to insufficient adaptation, while values above 0.1 cause instability. The meta-learning rate *β* governs the outer-loop update magnitude; it must be small to ensure stable convergence across heterogeneous tasks, typically in [0.001, 0.01]. The chosen values *α* = 0.01 and *β* = 0.001 follow standard MAML practice and were validated through preliminary experiments to balance adaptation speed and meta-training stability.

We report three complementary metrics; all computed on the query set after few-shot adaptation:

1. F1-score (F1): the harmonic mean of precision and recall for the positive class. $F1=2\times \left(\frac{Precision\;\times \;Recall}{Precision\;+\;Recall}\right)$
2. Balanced Accuracy (BA): the arithmetic mean of per-class recall values, $\;\mathrm{BA}=\frac{1}{2}\left({\mathrm{Recall}}_{\mathrm{normal}}+{\mathrm{Recall}}_{\mathrm{anomalous}}\right)$. This metric is insensitive to class distribution skew and is used as the primary metric in this study.
3. Macro F1-score (Macro F1): the unweighted average of per-class F1-scores, $\mathrm{Macro}\;F1=\frac{1}{2}\left({F1}_{\mathrm{normal}}+{F1}_{\mathrm{anomalous}}\right)$. Like Balanced Accuracy, it treats both classes equally regardless of their frequency.

In all experiments, the query set is class-balanced (57 normal + 57 anomalous samples). Nevertheless, we report both for completeness and consistency with the broader literature.

**Figure 3:**
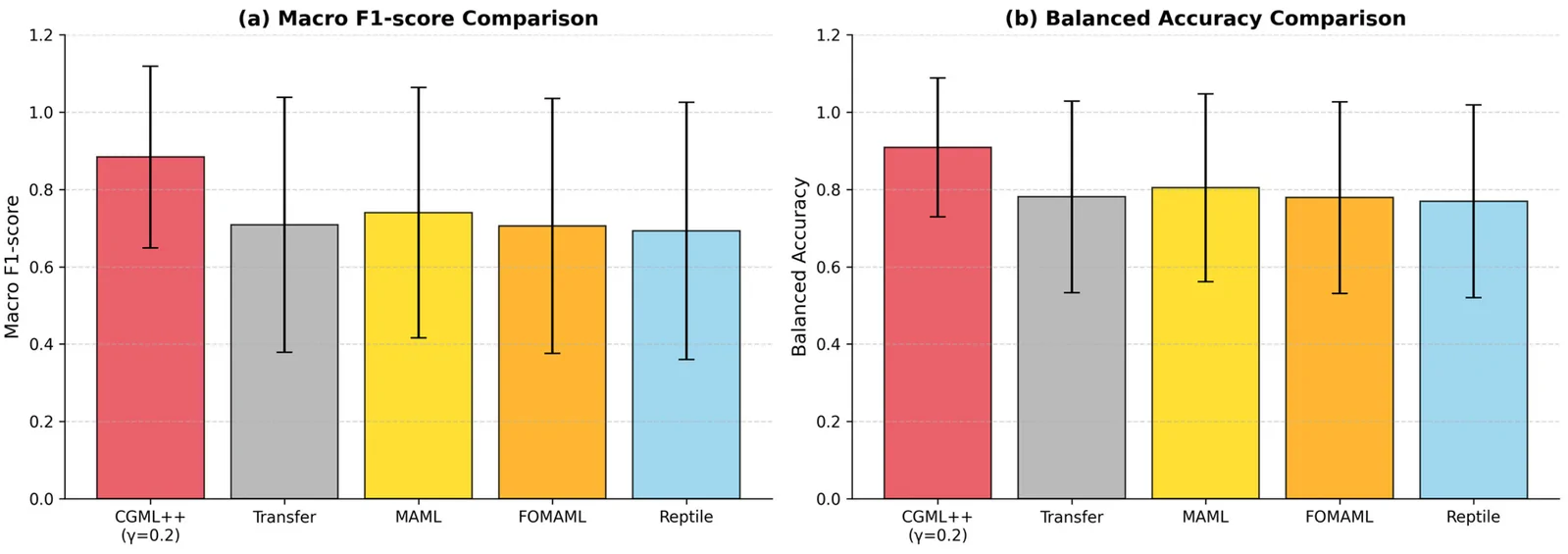
Performance comparison of CGML++ and baseline methods on the CWRU cross-sampling-rate task (12 kHz → 48 kHz, 1-shot) with ResNet1D backbone. (**a**) Macro F1-score; (**b**) Balanced Accuracy. Error bars represent standard deviation over 12 target sub-domains × 15 independent runs.

The interpolation parameter *γ* was selected based on the empirical scaling rule *γ*·*N* ≈ 2.0–3.5, which was discovered through a systematic analysis of the phase transition behavior (Section 3.3). Crucially, no target-domain labels are used for selecting *γ* in practice: the scaling rule depends only on the number of experts *N*, which is determined a priori by the source-domain partitioning. The *γ* sweep presented in Figure 3 was performed on a held-out validation split of the target domain solely for the purpose of visualizing the phase transition and validating the scaling rule; it is not part of the operational CGML++ pipeline. For a new target domain without any labeled data, a practitioner simply computes γ* ≈ 2.75/N and applies this value directly.

### 3.2. Comparison with Baseline Methods

#### 3.2.1. Cross-Frequency Transfer Results

We compare CGML++ against four established approaches: standard Transfer Learning (fine-tuning of a pre-trained model on the target domain), MAML [28], FOMAML [28], and Reptile [30]. All methods are trained on the same 12 source sub-domains (12 kHz) and evaluated under identical 1-shot conditions on the 48 kHz target sub-domains. To ensure a fair comparison, all baselines use the same backbone architectures, support/query splits, and number of adaptation steps as CGML++.

As shown in Table 3 and Figure 3, CGML++ consistently outperforms all baseline methods across every metric and all three architectures. We highlight the following observations:

CGML++ achieves the highest overall performance. On the ResNet1D backbone, CGML++ attains a Macro F1 of 0.8838 and a Balanced Accuracy of 0.9089, surpassing Transfer Learning by 0.1755 and 0.1280, respectively, and the strongest meta-learning baseline (MAML) by 0.1443 and 0.1047. Improvements are observed across all four architectures, with the most pronounced gains on CNN-based backbones (ResNet1D: +0.12 BA; LightCNN: +0.11 BA) and a smaller but consistent improvement on Transformer1D and Informer1D (+0.007 BA and +0.017 BA).

Standard Transfer Learning is insufficient under severe domain shift. Transfer Learning exhibits the weakest performance among all methods (Macro F1 = 0.7071), confirming that simple fine-tuning of a source-domain model cannot compensate for the fundamental signal representation change induced by the 4× sampling rate increase. This is consistent with the negative transfer phenomenon discussed in Section 1: the pre-trained features, optimized for 12 kHz statistics, become misaligned with the 48 kHz target distribution.

Meta-learning baselines improve over Transfer but remain limited. MAML, FOMAML, and Reptile all outperform Transfer Learning, demonstrating the value of learning a meta-learned initialization that is inherently more adaptable. However, their performance plateaus below CGML++ (e.g., MAML Macro F1 = 0.7395 vs. CGML++ 0.8838). We attribute this gap to the gradient conflict problem: when a single meta-model is trained across heterogeneous sub-domains (varying loads and defect severities), the aggregated gradients pull the initialization in conflicting directions. CGML++ avoids this by training independent experts and aggregating at the parameter level.

CGML++ exhibits superior stability. The standard deviation (for Macro F1) of CGML++ (0.2352) is consistently lower than those of MAML (0.3238) and Reptile (0.3323), indicating that the coarse-grained aggregation produces a more robust initialization that is less sensitive to the random sampling of support examples. This stability is critical for industrial deployment, where consistent performance across varying operating conditions is a prerequisite for trust in automated diagnostic systems.

From a practical standpoint, the compact cluster structure produced by CGML++ implies that the model’s predictions are more confident and less ambiguous, reducing the likelihood of borderline misclassifications in online monitoring scenarios.

#### 3.2.2. Cross-Load Transfer Results

To assess the generalizability of CGML++ across different types of domain shift, we additionally evaluate all methods under the cross-load transfer scenario (HP 0–1–2 → HP 3, 1-shot, binary classification). The domain shift metrics (Table 1) confirm that this configuration exhibits the highest MMD (0.151) among all cross-load splits, yet remains substantially below the cross-frequency shift (MMD = 0.189).

**Figure 4:**
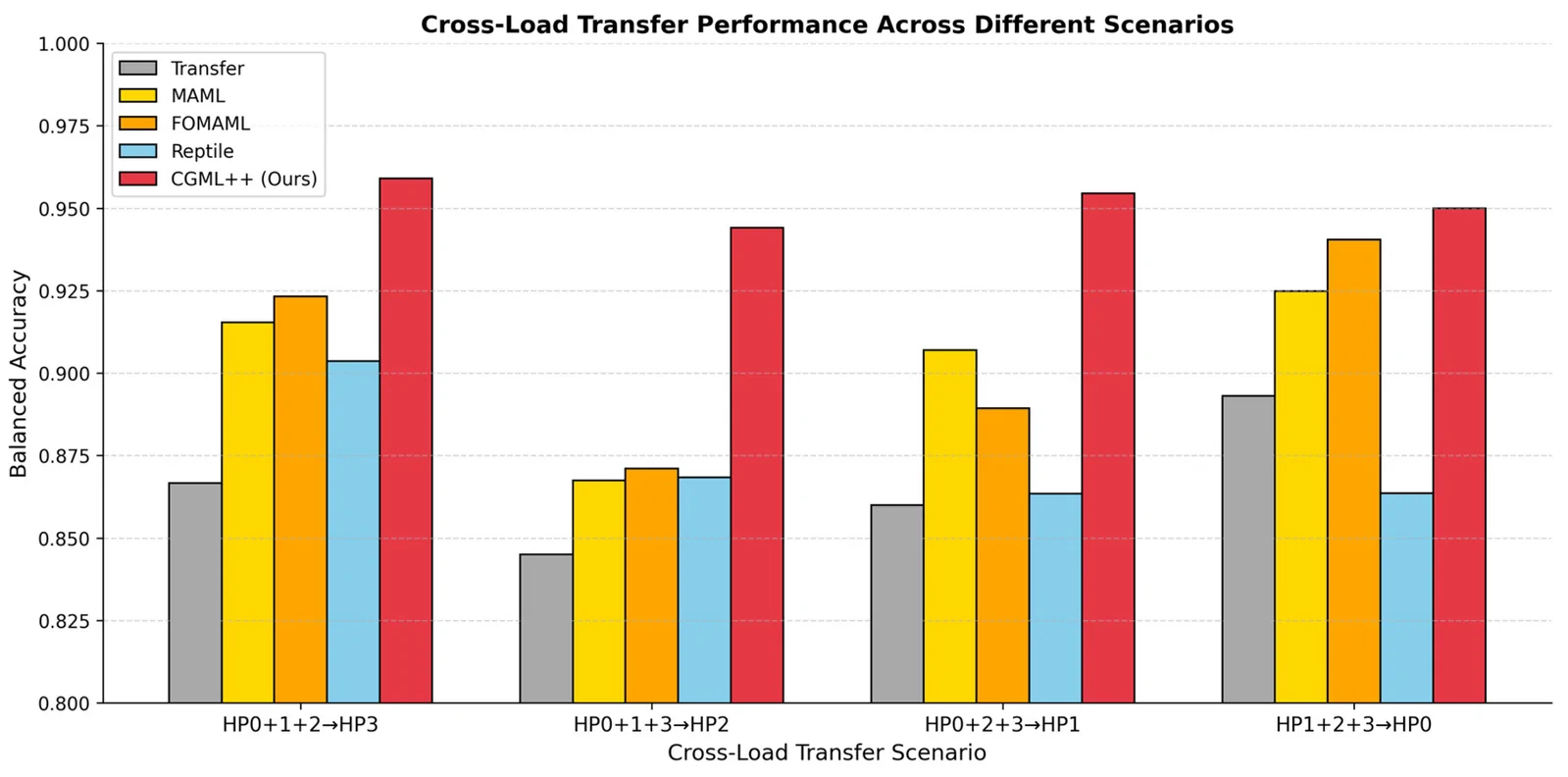
Balanced accuracy comparison of CGML++ and baseline methods across four cross-load transfer scenarios (ResNet1D backbone, 1-shot). CGML++ consistently outperforms all baselines across all scenarios, demonstrating robustness to different source-target load configurations.

Figure 4 and Table 4 summarizes the results. CGML++. The phase transition in *γ* is also observed under cross-load, with the optimal γ* ≈ 0.05, corresponding to *γ*·*N* ≈ 0.45. Notably, the optimal *γ*·*N* for cross-load is lower than for cross-frequency (0.45 vs. 2.0–3.5), consistent with the milder domain shift: less expert contribution is needed when the transfer model already provides a near-optimal initialization.

#### 3.2.3. Cross-Architecture Results

**Figure 5:**
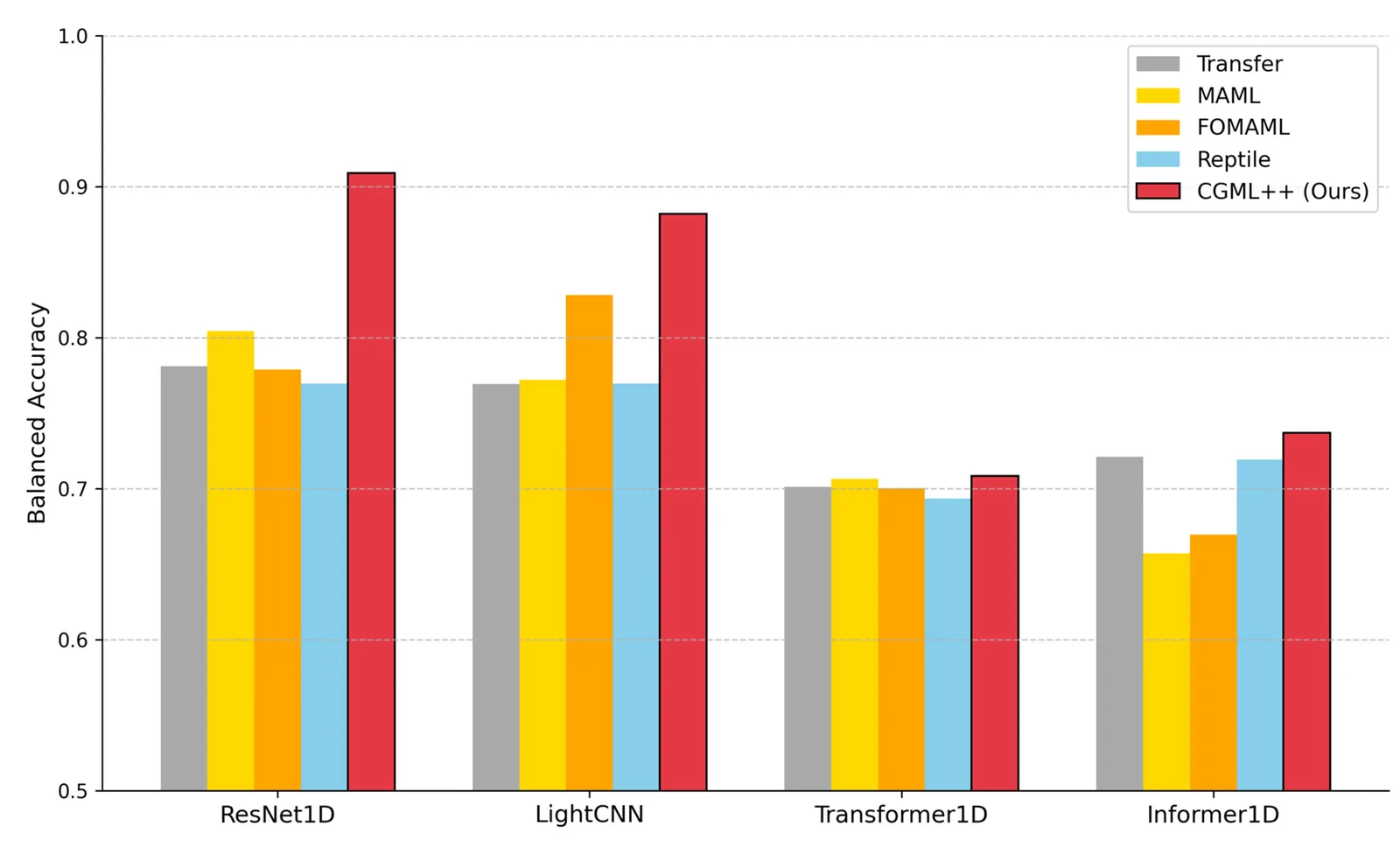
Balanced accuracy comparison of CGML++ and baseline methods across four neural architectures (ResNet1D, LightCNN, Transformer1D, Informer1D) under cross-sampling-rate transfer (12 kHz → 48 kHz, 1-shot).

The superiority of CGML++ is consistent across all four backbone architectures. Notably, the performance gap between CGML++ and the baselines is preserved even on LightCNN (~50K parameters), demonstrating that the benefits of coarse-grained aggregation are not contingent on model capacity. This architectural agnosticism suggests that CGML++ can be deployed on resource-constrained industrial edge devices without sacrificing diagnostic accuracy. Results for cross-architecture comparison are shown in Table 5 and on Figure 5.

### 3.3. Analysis of Parameter γ and Phase Transition

A key empirical contribution of this work is the discovery of a phase transition in the behavior of CGML++ as a function of the aggregation parameter γ. To characterize this effect, we systematically varied γ across the full admissible range [0, 0.5] and evaluated the resulting Macro F1 on all 12 target sub-domains.

**Figure 6:**
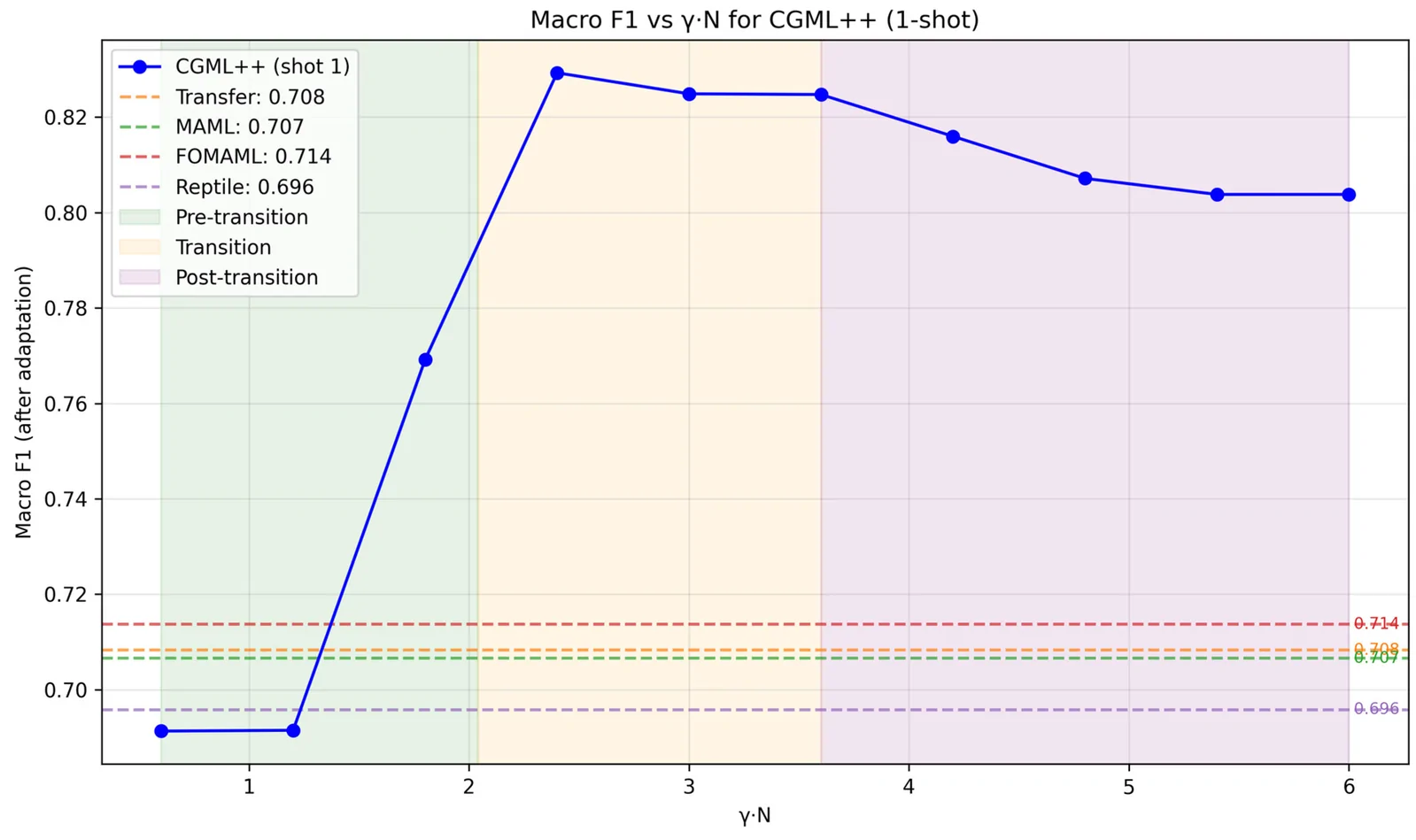
Macro F1-score as a function of the effective interpolation coefficient *γ*·*N*, averaged over all 12 target sub-domains (ResNet1D backbone). Horizontal dashed lines mark the performance of Transfer Learning, MAML, Reptile, and FOMAML. The highlighted region (*γ*·*N* ∈ [2.0, 3.5]) corresponds to the phase transition zone where classification performance improves sharply.

As illustrated in Figure 6, the performance of CGML++ exhibits a distinctive three-regime behavior:

Regime 1: Pre-transition (γ·N≲1.5). The expert contribution is too small to restructure the initialization; it merely injects source-specific bias. Performance stays at or marginally below Transfer Learning (Macro F1 ≈ 0.69 vs. 0.71).

Regime 2: Phase transition and optimal plateau (γ·N≈2.0–3.5). The Macro F1 rises sharply, crossing all baselines at γN≈1.7–1.8 and peaking at γN≈2.4 (Macro F1 ≈ 0.83), with a broad, flat plateau up to γN≈3.6. The width of this plateau makes the method tolerant to the exact choice of γ.

Regime 3: Extrapolation overshoot (γ·N≳3.5). The formula extrapolates increasingly far past the averaged experts; performance declines gently as source bias is amplified. Crucially, even deep in this regime (γN=6) CGML++ (0.80) still outperforms every baseline (best 0.77), confirming strong robustness to γ mis-specification.

The borderline values *γ*·*N* = 1.5, 2.0, and 3.5 are empirical thresholds identified from the performance curve (Figure 6), rather than theoretically derived constants. This phase transition reveals a practical scaling rule: optimal performance is consistently achieved when γ·N≈2.0–3.5, across all 12 target sub-domains examined in this study. This empirical rule significantly simplifies hyperparameter selection in practice. Rather than performing an exhaustive grid search over γ, a practitioner need only compute $\gamma^{*}\approx 2.0/N$ (the midpoint of the transition zone) based on the number of experts N, which is determined a priori by the source domain partitioning. For N=12, this yields $\gamma^{*}\approx 0.167$, requiring no validation data from the target domain.

## 4. Discussion

### 4.1. Interpretation of Results

The experimental results presented in Section 3 demonstrate that CGML++ consistently outperforms both transfer learning and standard meta-learning approaches under a severe domain shift (12 kHz → 48 kHz) in the 1-shot setting. We now discuss the underlying reasons for this superior performance.

Transfer Learning relies on fine-tuning a single model pre-trained on the source domain. Under a severe domain shift—such as the fourfold change in sampling rate from 12 kHz to 48 kHz—the source-learned features may not generalize to the target distribution. Fine-tuning with only one labeled example per class frequently leads to overfitting to the support set or destructive interference with previously learned source features, as the model lacks sufficient signal to distinguish target-specific patterns from noise. In contrast, CGML++ combines the transfer-initialized model with multiple domain-specific experts, each capturing distinct vibration patterns from different sub-domains. This aggregation provides a richer and more robust initialization for few-shot adaptation, reducing the risk of negative transfer. Self-supervised pre-training methods such as TS2Vec [41] offer an alternative approach to learning robust representations without labeled data, and could be combined with CGML++ to further improve initialization quality.

Standard meta-learning algorithms learn a single initialization by averaging gradients across tasks. While effective for homogeneous task distributions, this approach struggles with heterogeneous domains where task-specific gradients point in conflicting directions. In the CWRU dataset, different loads and defect severities produce distinctly different vibration signatures, leading to gradient conflicts during meta-training that degrade the quality of the learned initialization. CGML++ circumvents this issue by training independent MAML experts on each sub-domain and aggregating their weights rather than their gradients. This preserves the specialization of each expert while enabling knowledge sharing through parameter-level averaging. The parametrized coefficient γ further allows the method to balance global knowledge (from the transfer model) with local expertise (from the averaged experts), adapting the interpolation strength to the characteristics of the target domain.

The role of γ and the phase transition. The observed phase transition at γ·N≈2.0–3.5 provides insight into the dynamics of coarse-grained aggregation:

- When γ·N≲1.5, the transfer model dominates the initialization, and performance remains close to standard Transfer Learning.
- When γ·N≈2.0–3.5, the contributions of the transfer model and the averaged experts reach a productive balance, yielding optimal adaptation.
- When γ·N≳3.5, the extrapolation extends too far past the averaged experts, amplifying source-domain bias and degrading generalization.

This behavior is reminiscent of phase transition phenomena observed in other machine learning contexts [33], where optimal generalization occurs at a critical balance between competing sources of information. The sharpness of the transition suggests that the parameter space of the aggregated model undergoes a qualitative restructuring within this narrow range, transforming a poorly aligned initialization into one that is inherently conducive to few-shot adaptation. Notably, the optimum requires extrapolating past the averaged experts (γN>1) rather than interpolating toward them, indicating that vanilla expert averaging provides only a midpoint along the path to the optimal initialization.

### 4.2. The Scaling Rule and Its Practical Implications

A key empirical contribution of this work is the discovery of a scaling rule relating the optimal value of γ to the number of experts N. Specifically, we find that optimal performance is consistently achieved when γ·N≈2.0–3.5. This empirical rule has significant practical implications. Instead of performing an expensive grid search over γ, practitioners can estimate the optimal value directly from N, which is determined a priori by the source domain partitioning. For example, with N=12 experts (as in this work), the optimal γ lies in the range $\left[0.17,0.30\right]$, with the midpoint $\gamma^{*}\approx 0.20$ serving as a reliable default. This reduces hyperparameter tuning effort considerably—a property particularly valuable in industrial applications where computational resources and labeled target data are limited.

Moreover, the scaling rule implies that the optimal operating point depends only on the product γ·N, not on the individual values of γ and N. This suggests that the method generalizes to different numbers of experts without requiring extensive re-tuning: doubling N simply halves the optimal γ, preserving the same effective interpolation strength. This scalability makes CGML++ practical for deployment across diverse industrial settings with varying numbers of available operating conditions.

An important observation emerges from comparing the two domain shift scenarios: the optimal value of *γ* differs substantially between cross-frequency (γ* ≈ 0.23, *γ*·*N* ≈ 2.0–3.5) and cross-load (γ* ≈ 0.05, *γ*·*N* ≈ 0.45). This reveals that the scaling rule *γ*·*N* = const is not universal but shift-dependent: the constant itself varies with the severity of the domain shift. Specifically, we hypothesize that the optimal *γ*·*N* scales monotonically with the MMD between source and target distributions. For severe structural shifts (high MMD, e.g., cross-frequency, MMD = 0.189), a large expert contribution is needed (*γ*·*N* ≈ 2.0–3.5). For mild amplitude shifts (low MMD, e.g., cross-load, MMD = 0.151), the transfer model already provides a near-optimal initialization, and a small expert correction suffices (*γ*·*N* ≈ 0.45). We therefore define the applicability scope of the scaling rule as follows: the range *γ*·*N* ∈ [2.0, 3.5] applies to domain shifts characterized by fundamental changes in signal representation (e.g., cross-sampling-rate, cross-sensor). For milder shifts involving only amplitude or frequency modulation without representation change, the optimal *γ*·*N* is expected to fall in the range [0.3, 0.6]. A formal characterization of this relationship (e.g., via regression on MMD) is left as future work.

### 4.3. Architectural Agnosticism

We validated CGML++ on four fundamentally different neural architectures: ResNet1D (2.8M parameters, residual connections), LightCNN (50K parameters, lightweight convolutions), Transformer1D (2.5M parameters, full self-attention) and Informer1D (0.94M parameters, ProbSparse attention with convolutional distilling). Future work could extend this analysis to frequency-domain Transformers such as FEDformer [42], which may offer complementary advantages for vibration signal analysis. All four architectures benefited consistently from CGML++ aggregation.

The fact that CGML++ operates effectively across such diverse architectures confirms that the proposed aggregation strategy is not tied to specific inductive biases or model design choices. Instead, it operates at the level of parameter space, making it broadly applicable to any differentiable model whose parameters can be meaningfully averaged.

### 4.4. Limitations

Despite its strengths, CGML++ has several limitations that should be acknowledged:

- Computational cost. Training N independent MAML experts requires approximately N times more computational resources than training a single model. In this work, N=12, which is manageable on modern GPU hardware, but for applications with a larger number of sub-domains, the cost may become prohibitive. Techniques such as expert distillation, partial weight sharing, or parallel training could mitigate this overhead.
- Dependence on expert quality. The aggregated initialization is only as good as its constituent experts. If one or more experts are poorly trained—due to insufficient data, label noise, or suboptimal hyperparameters—the uniform averaging may degrade the final initialization. Robust aggregation mechanisms, such as outlier rejection or performance-weighted averaging, could address this vulnerability but are not explored in the current work.
- Evaluation scope. Our experiments are limited to the 1-shot binary classification setting on a single dataset (CWRU), evaluated under two domain shift scenarios (cross-sampling-rate and cross-load). While we expect the method to generalize to higher-shot scenarios (5-shot, 10-shot) and multi-class fault diagnosis, this has not been empirically validated. Furthermore, although CWRU is the most widely used benchmark in bearing fault diagnosis, generalization to other datasets (e.g., PU, MFPT, XJTU-SY) and to real industrial data with higher noise levels remains to be demonstrated.
- Empirical nature of the scaling rule. The scaling rule γ·N≈2.0–3.5 is an empirical observation, not a theoretically derived result. While it holds consistently across all 12 target sub-domains and three architectures in this study, a formal theoretical characterization of the phase transition remains an open question.

### 4.5. Future Work

Several promising directions emerge from this work:

- Multi-class fault classification. Extending CGML++ to distinguish between individual fault types (inner race, outer race, ball, and combined defects) would significantly broaden its applicability in real-world diagnostic systems.
- Automatic γ selection. Developing a mechanism to estimate the optimal γ based on measurable domain similarity metrics (e.g., maximum mean discrepancy between source and target features) would eliminate the need for any manual tuning. Our domain shift analysis (Table 1) suggests that MMD, rather than A-distance, is the most informative metric for this purpose, as it correlates with actual model performance degradation under domain shift, whereas A-distance primarily captures linear separability of source and target samples.
- Computational optimization. Exploring expert distillation, progressive training, or early stopping strategies could reduce the cost of training multiple experts without sacrificing aggregation quality.
- Theoretical analysis. A formal investigation of the phase transition—potentially through the lens of loss landscape geometry [35] or neural tangent kernel theory [43]—could provide deeper insights into the dynamics of coarse-grained aggregation and guide the design of future meta-learning strategies.
- Patch-based Transformers. Exploring patch-based Transformer architectures such as PatchTST [44], which have shown strong performance on time-series classification tasks, could further validate the architectural agnosticism of CGML++.

## 5. Conclusions

In this paper, we presented CGML++ (Coarse-Grained Meta-Learning++), a novel few-shot learning method for binary anomaly classification in bearing fault diagnosis under severe domain shifts. Our approach extends the coarse-grained meta-learning paradigm by introducing a parametrized aggregation of MAML-trained domain experts, combined with a transfer-initialized model through a tunable coefficient *γ*.

We evaluated CGML++ on the CWRU bearing dataset under two domain shift scenarios—cross-sampling-rate transfer (12 kHz → 48 kHz) and cross-load transfer—in a 1-shot setting. The experimental results demonstrate that CGML++ consistently outperforms standard Transfer Learning, MAML, Reptile, and FOMAML across all evaluation metrics. Specifically, CGML++ achieves a Macro F1-score of 0.88, surpassing Transfer Learning by 0.18 and the best-performing meta-learning baseline by 0.15. Under cross-load transfer, CGML++ achieves a Balanced Accuracy of 0.9590, confirming its effectiveness across different types of domain shift. The optimal aggregation parameter is shift-dependent (*γ*·*N* ≈ 2.0–3.5 for cross-frequency, *γ*·*N* ≈ 0.45 for cross-load), revealing that the scaling rule adapts to the severity of the domain shift as quantified by MMD.

A key contribution of this work is the discovery of a phase transition in the behavior of the aggregation parameter *γ*. We empirically observed that optimal performance is achieved when *γ*·*N* ≈ 2.0–3.5, where *N* is the number of experts. This finding leads to a practical scaling rule that significantly simplifies hyperparameter selection, eliminating the need for exhaustive grid search. The scaling rule holds across different target domains, suggesting that the product *γ*·*N* is the fundamental quantity governing the balance between global knowledge (from the transfer model) and local expertise (from the averaged experts). We further demonstrated the architectural agnosticism of CGML++ by validating it on four distinct neural architectures: ResNet1D, LightCNN, Transformer1D and Informer1D. The consistent improvements over baselines across all architectures confirm that CGML++ is a general-purpose framework applicable to a wide range of time-series classification tasks.

Future work will focus on extending the method to multi-class fault classification, testing on additional bearing datasets (PU, MFPT, XJTU-SY), and developing automatic *γ* selection mechanisms based on domain similarity metrics. Additionally, we plan to investigate the theoretical foundations of the observed phase transition and scaling rule, which could provide deeper insights into the dynamics of coarse-grained meta-learning.

In summary, CGML++ offers a robust, scalable, and practical solution for few-shot fault diagnosis under severe domain shifts, with potential applications in real-world industrial condition monitoring systems.

## Figures and Tables

**Table 1:** Domain shift metrics for cross-load and cross-frequency setups.

Source Domain	Target Domain	A-Distance	MMD	Spectral Distance
All 12 domains 12 kHz	All 12 domains 48 kHz	1.949	0.189	1.322
HP0 + HP1 + HP2	HP3	1.738	0.151	0.980
HP0 + HP1 + HP3	HP2	0.782	0.030	0.917
HP0 + HP2 + HP3	HP1	0.824	0.026	0.909
HP1 + HP2 + HP3	HP0	1.920	0.061	1.106

**Table 2:** Hyperparameter settings for CGML++ and baselines.

Hyperparameter	Symbol	Value
Inner learning rate	*α*	0.01
Meta learning rate	*β*	0.001
Adaptation learning rate	*α′*	0.001
Inner-loop steps	*K*	5
Meta-iterations	*M*	10
Interpolation parameter range	*γ*	[0, 0.5]
Number of experts	*N*	12
Window size	*-*	1024
Batch size (meta-training)	*B*	64
Number of independent runs	*-*	15
Optimizer	*-*	Adam
Hardware	*-*	RTX 3090
Python version	*-*	3.10

**Table 3:** Performance comparison of CGML++ and baseline methods on the CWRU cross-sampling-rate task (12 kHz → 48 kHz, 1-shot) on ResNet1D architecture. Values are reported as mean ± standard deviation over 12 target sub-domains × 15 independent runs. Best results per column are highlighted in bold.

	CGML++ *γ* = 0.2	Transfer Learning	MAML	FOMAML	Reptile
F1-score	**0.9089 ± 0.2082**	0.7146 ± 0.3898	0.7545 ± 0.3681	0.7106 ± 0.3917	0.6907 ± 0.4037
Balanced accuracy	**0.9089 ± 0.1797**	0.7809 ± 0.2475	0.8042 ± 0.2428	0.7788 ± 0.2475	0.7694 ± 0.2492
Macro F1 score	**0.8838 ± 0.2352**	0.7083 ± 0.3298	0.7395 ± 0.3238	0.7056 ± 0.3299	0.6926 ± 0.3323

**Table 4:** Balanced accuracy comparison of CGML++ and baseline methods on the CWRU cross-load tasks on ResNet1D architecture.

Source → Target	CGML++	Transfer Learning	MAML	FOMAML	Reptile
HP0 + HP1 + HP2 → HP3	0.9590 ± 0.1109	0.8666 ± 0.1302	0.9153 ± 0.1103	0.9232 ± 0.1142	0.9036 ± 0.1168
HP0 + HP1 + HP3 → HP2	0.9440 ± 0.1043	0.8450 ± 0.1292	0.8674 ± 0.1296	0.8711 ± 0.1308	0.8684 ± 0.1166
HP0 + HP2 + HP3 → HP1	0.9545 ± 0.0992	0.8600 ± 0.1105	0.9069 ± 0.1004	0.8893 ± 0.1160	0.8634 ± 0.1255
HP1 + HP2 + HP3 → HP0	0.9500 ± 0.1184	0.8931 ± 0.1372	0.9248 ± 0.1351	0.9404 ± 0.1084	0.8635 ± 0.1923

**Table 5:** Balanced accuracy comparison of CGML++ and baseline methods on ResNet1D, LightCNN, Transformer1D and Informer1D architectures.

	CGML++	Transfer Learning	MAML	FOMAML	Reptile
ResNet1D	0.9089	0.7809	0.8042	0.7788	0.7694
LightCNN	0.8819	0.7693	0.7720	0.8282	0.7694
Transformer1D	0.7084	0.7010	0.7063	0.7001	0.6933
Informer1D	0.7370	0.7209	0.6572	0.6696	0.7192

## Data Availability

The data presented in this study are available on request from the corresponding author.
